# Review of West Nile virus circulation and outbreak risk in Madagascar: Entomological and ornithological perspectives

**DOI:** 10.1051/parasite/2016058

**Published:** 2016-11-16

**Authors:** Michaël Luciano Tantely, Steven M. Goodman, Tsirinaina Rakotondranaivo, Sébastien Boyer

**Affiliations:** 1 Medical Entomology Unit, Institut Pasteur de Madagascar, Ambatofotsikely BP 1274 Antananarivo 101 Madagascar; 2 Field Museum of Natural History 1400 South Lake Shore Drive Chicago 60605 Illinois USA; 3 Association Vahatra BP 3972 Antananarivo 101 Madagascar

**Keywords:** Mosquitoes, Vector status, West Nile virus, Migratory birds, Madagascar

## Abstract

West Nile fever (WNF) is a zoonotic disease, occurring nearly globally. In Madagascar, West Nile virus (WNV) was first detected in 1978 from wild birds and the virus is currently distributed across the island, but no epidemic or epizootic period has been recorded. One fatal human case of WNV infection was reported in 2011, suggesting a “tip of the iceberg” phenomenon of a possible WNF epidemic/epizootic on the island. The main objective of this literature-based survey is to review patterns of WNV circulation in Madagascar from the entomological and ornithological points of view. Among the 235 mosquito species described from Madagascar, 29 species are widely associated with WNV infection; 16 of them are found naturally infected with WNV on the island and categorized into major, candidate, and potential vectors of WNV according to their vector capacity. This study upholds the hypothesis that WNV enzooticity is independent of annual movements of migratory birds passing through Madagascar. Moreover, the lack of regular migratory bird flux between Africa and Madagascar would reduce the probability of transmission and the subsequent reintroduction of the virus into locally occurring mosquito species. Given that Palearctic migratory birds are strongly implicated in the transmission of WNV, we highlight notable differences in the movements and species diversity of these birds in Madagascar as compared to eastern and northern Africa. Risk factors from this two-pronged approach are presented for the emergence of WNF outbreak.

## Abstract (fr)

La Fièvre du Nil Occidental (FNO) est une zoonose qui sévit presque partout dans le monde. À Madagascar, la première détection du Virus du Nil Occidental (VNO) remonte à 1978 chez des oiseaux sauvages et le virus est actuellement réparti à travers l’île, mais aucune période épidémique ni épizootique n’a été signalée. Un cas mortel associé à l’infection par le VNO signalé en 2011 suggère une « pointe de l’iceberg » d’une possible épidémie ou épizootie dans l’île. L’objectif principal de cette revue basée sur la littérature est d’examiner du point de vue entomologique et ornithologique la circulation du VNO à Madagascar. Parmi les 235 espèces de moustiques répertoriées à Madagascar, 29 espèces sont déjà associées au VNO ; 16 d’entre elles ont été trouvées naturellement infectées par le VNO et peuvent être groupées en vecteurs majeurs, candidats et potentiels selon leurs capacités vectorielles. Cette étude rapporte une circulation enzootique du VNO, indépendante des mouvements annuels des oiseaux migrateurs qui passent par Madagascar. De plus, l’absence de flux régulier d’oiseaux migrateurs entre l’Afrique et Madagascar réduirait la probabilité de la transmission et la réintroduction du VNO chez les espèces de moustiques présentes localement. Étant donné que les oiseaux migrateurs paléarctiques sont fortement impliqués dans la transmission du VNO, nous mettons en évidence des différences notables dans les mouvements et la diversité des espèces de ces oiseaux à Madagascar par rapport à l’Afrique de l’Est et du Nord. Les facteurs de risque de cette approche à deux volets sont présentés pour l’émergence d’épidémie de FNO.

## Introduction

West Nile fever (WNF) is a zoonotic disease affecting different vertebrates, such as birds, mammals, rodents, humans, non-human primates, reptiles, and amphibians [10]. The disease is caused by an arthropod-borne virus belonging to the genus *Flavivirus*, family Flaviviridae [18].

The virus causing WNF, known as West Nile virus (WNV), was first isolated in 1937 from a woman with high fever living in the West Nile District of Uganda [120]. The first outbreak was reported from Israel in 1951–1952 [13]. Subsequently, WNF has been documented across the planet with the exception of Antarctica [17, 63, 72, 87, 90, 118].

Eight lineages of WNV have been identified based on the sequences of structural protein genes of lineages 1–5 [28, 41, 71, 90, 137]. Lineages 1 and 2 consist of different WNV strains isolated from field collections [41] and are pathogenic in wild and domestic animals and humans [49, 69, 116, 138]. Lineage 1 is distributed virtually worldwide and is frequently associated with WNF outbreaks in humans [62]. Lineage 2 is maintained in sub-Saharan African and Madagascan enzootic foci [90, 96]. This lineage has been demonstrated to cause human outbreaks in Europe and South Africa [16, 91] and poses a continuing threat to other regions around the world [77].

In Madagascar, WNV is more abundant and widely spread than two others *Flaviviruses* (Dengue and Dakar Bat virus) known to circulate on the island [34]. In the same *Flavivirus* genus, the circulation of Zika and Yellow fever has not yet been reported in Madagascar (at least up to September 2016), but they have recently been considered a Malagasy public health emergency. WNV isolation in Madagascar was performed on inoculated mosquito cell cultures (C6/36 or AP-61) and newborn mice [34]. The first isolation of the virus from wild birds was in 1978, which involved egrets of the genus *Egretta* (family Ardeidae) and endemic parrots *Coracopsis vasa* (family Psittacidae) [76]. The virus was later isolated from pooled mosquitoes and febrile humans [34, 35, 75]. A serological survey was carried out using a hemagglutination-inhibition assay (HAI) [23], a monoclonal antibody-based capture Enzyme-Linked Immunosorbent Assay (MAb-based capture ELISA) [53], and recently by ID Screen West Nile Competition Multi-species ELISA that is coupled with semi-nested reverse transcription polymerase chain reaction (RT-PCR) on WNV nucleic acid [75]. On the island, the first WNV antibodies were detected in 1953 [122]. Subsequently, WNV antibodies have been detected in humans [34, 68], cattle, bats, lemurs, and rodents [26, 34]. Lineage 2 is known to circulate in Madagascar, in Eastern and Southern Africa, in Europe and Eastern Europe, and in Russia [12, 41, 75, 98, 118]. No information is available regarding WNV infection in Malagasy horse populations.

To date, neither epidemic nor epizootic episodes have been reported in Madagascar, despite serological and virological data that demonstrate widespread circulation of WNV across 18 districts distributed in all the bioclimatic domains of the island [34, 68, 75, 76, 84]. Examples of circulation in humans include a case of encephalitis associated with WNV infection in 2001 in an adult and children hospitalized in Antananarivo [78] and one fatal case of WNV infection reported in a tourist ten years later after a visit to the lowland western wetland of Lac Kinkony and return to La Réunion [64]. No explanation exists in the literature to account for the broad distribution of WNV in Madagascar without significant outbreaks. This study provides a review from both the entomological and ornithological perspectives, and aims to help explain observed patterns of WNV circulation in Madagascar.

## WNV infection in mosquitoes, birds, and other vertebrates

In a worldwide context, the first evidence of WNV transmission *via* bites from infected mosquitoes was found in 1943 in a laboratory experiment, with the vector being *Aedes albopictus* [97].

It has been determined that 10^1.5^ plaque-forming units (PFU)/mL is the minimum viremia titer in infectious birds known to infect adult mosquitoes orally (*Aedes triseriatus*, *Ae. vexans*, and *Culex pipiens*) [99]. Individual mosquitoes remain infected throughout their life [110]. The length of the extrinsic incubation period decreases with increasing ambient temperature [109]: the extrinsic cycle in mosquitoes could last three to five days at 26 °C to 30 °C and 36 days at 14 °C after taking the infected blood meal [2, 109]. The incubation period is also influenced by the presence of a mesenteronal infection barrier [21], a mesenteronal escape barrier [60], a salivary gland infection barrier [46], and a salivary gland escape barrier [42, 60, 92]. WNV vertical transmission has been described under field conditions in Kenya for *Culex univittatus* [79] and in East Baton Rouge Parish, Louisiana for *Cx*. *salinarius*, and *Ae*. *triseriatus* [135] and under laboratory conditions for *Ae*. *aegypti*, *Ae*. *albopictus*, *Cx*. *pipiens*, *Cx*. *quinquefasciatus*, *Cx. tarsalis*, and *Cx*. *tritaeniorhynchus* [3, 11, 39]. Persistence of WNV in overwintering mosquitoes under field conditions has been reported for *Cx*. *pipiens* [86].

Excluding non-vector routes of WNV transmission such as direct contact with infected blood, tissues, aerosols, feces [10], human breast milk [51], and transplacental transmission [114], mosquito infection is obligatory to complete the WNV transmission cycle, and to transmit the virus to vertebrate hosts, including humans [109]. The minimum viremia titer (10^1.5^ PFU/mL) able to infect mosquitoes can occur 12 hr after virus inoculation in the following bird orders: Passeriformes, Falconiformes, Charadriiformes, Strigiformes, Anseriformes, Piciformes, Columbiformes, Psittaciformes, and Galliformes, and 36 hr in Gruiformes [59]. It can be maintained for four days for Piciformes and Columbiformes, beyond seven days for Passeriformes and Charadriiformes, and between five and seven days for the remaining orders listed above [59].

Experimental infection performed on different mammals and Crocodilia reported this level of viremia to be sufficient to infect mosquitoes [10, 57, 136]. However, most infected mammals have short lasting viremia and others exhibit viremia that is age-dependent (i.e. observed in young animals) [10, 57]. These different aspects need to be taken into account during epizootic periods [10]. Humans can develop viremia able to infect mosquitoes but they are still considered as dead-end hosts in the WNV transmission cycle [10].

## Mosquito WNV vectors in Madagascar

In Madagascar, 29 mosquito species of the 235 (12%) described culicid fauna [127] are known in a worldwide context to be associated with WNV infection (Table 1). These species belong to five genera (*Aedeomyia*, *Aedes*, *Anopheles*, *Culex*, and *Mansonia*). Of these 29 mosquito species, 25 are not native to Madagascar and they can be categorized according to three criteria (natural infection, vector competence, and field vector-host contact) to classify the vector status of a given species [125].

**Table 1.** Biology, vector competence, and vector status of Malagasy mosquito species naturally/experimentally associated with WNV in Madagascar.

Species	Natural infection (Ni)			Biology		Vector competence (Vc)				Vector statuts	References		
	Locality	Date	Biotope^µ^	FB	Density	IR	Dose	TR	Dose		Ni	Biology	Vc
***Ad. madagascarica***	Madagascar	Nov-12	Lake	O	LAb					CV	[75]	[15]	
***Ae. albocephalus***	Madagascar	Dec-82	Forest	GF	LAb					CV	[34]	[34, 106]	
*Ae. dalzieli*	Senegal			GF	R					PV	[1]	[127]	
***Ae. madagascarensis***	Madagascar	July-83	Forest	A	R					PV	[34]	[34]	
***Ae. circumluteolus***	Madagascar	Dec-82	Forest	A	LAb					CV	[34]	[65, 106, 127]	
***Ae. aegypti***	Madagascar	Nov-82	Forest	A	LAb	16%	10^7.2±0.3^ PFU/mL	≤16%	10^7.2±0.3^ PFU/mL	CV	[34]	[4, 34]	[134]
*Ae. albopictus*	USA			A*	Ab	90%	10^7.2±0.3^ PFU/mL	73%	10^7.2±0.3^ PFU/mL	CV	[135]	[34]	[134]
***An. coustani***	Madagascar	Nov-12	Lake	GF*	Ab					CV	[75]	[127]	
***An. brunnipes***	Madagascar	May- 88	Village	A	R					PV	[34]	[124]	
***An. maculipalpis***	Madagascar	Dec-85	Village	GF	LAb					PV	[34]	[34]	
***An. pauliani***	Madagascar	Jun-13	Village	GF	LAb					PV	[75]	[14]	
*Cq. metallica*	Uganda			A	R					PV	[116]	[66, 127]	
*Cx. poicilipes*	Senegal			GF	Ab					PV	[131]	[127]	
***Cx. antennatus***	Madagascar	Mar-88	Village	GF*	Ab					CV	[34]	[127]	
***Cx. decens***	Madagascar	Feb, April, Dec-85	Forest edge	GF*	Ab					CV	[34]	[34, 129]	
*Cx. guiarti*	Ivory Coast			A	R					PV	[1]	[34]	
*Cx. neavei*	Sengal			GF	R					PV	[131]	[32]	
*Cx. pipiens*	USA			GF	LAb	17–100%	10^5.2 ±0.2^–10^7.1±0.1^ PFU/mL	2–33%	10^5.2 ±0.2^–10^7.1±0.1^ PFU/mL	CV	[20]	[127]	[38, 134]
***Cx. quinquefasciatus***	Madagascar	Feb-86	Forest egde	GF*	Ab	8–86%	10^7.1±0.1^ PFU/mL	2–52%	10^7.1±0.1^ PFU/mL	MV	[34]	[34]	[38]
***Cx. scottii*** +	Madagascar	July-83	Village	A	R					PV	[34]	[34]	
***Cx. tritaeniorhynchus***	Madagascar	Dec-82	Forest	GF*	Ab	10–90%	10^0.79^–10^2.87^ SMICLD50/mL	100%	10^−1.06^ SMICLD50/mL	MV	[34]	[14]	[48]
***Cx. univittatus***	Madagascar	Feb-86	Forest egde	GF	Ab	51%	10^5.8–7.2^ PFU/mL	100%	7.0 log 10 CPD50/mL	MV	[34]	[127]	[25, 70]
***Cx. vansomereni***					R	42%	10^5.8–7.2^ PFU/mL		17–100%			[129]	[70]
*Cx. weschei*	CAR			A	R					PV	[24]	[34]	
*Lt. tigripes*	CAR				R					PV	[116]	[129]	
*Ma. africana*	Senegal			A	R	50%	10^5.8–7.2^ PFU/mL			PV	[1]	[34]	[70]
***Ma. uniformis***	Madagascar	June-13	Lake	GF*	Ab	43%	10^5.8–7.2^ PFU/mL			MV	[75]	[75]	[70]
*Mi. hispida*	Senegal			A	R					PV	[131]	[127]	
*Mi. splendens*	Senegal			unknown	R					PV	[131]	[127]	

In bold are mosquitoes found naturally infected with WNV in Madagascar.

+ This species was not specified and is morphologically close to *Culex scottii* [34].

Ni: natural infection, Locality: place where a mosquito was found naturally WNV-positive, USA: United States of America, CAR: Central African Republic. Date: periods of WNV detection in Madagascar in mosquitoes, Biotope: biotope where the mosquitoes found WNV-positive were collected, Lake: village around lake.

FB: feeding behavior, O: ornithophilic, Z: zoophilic (ruminants), A: anthropophilic, GF: general feeder (Z, O, A).

Ab: abundant, LAb: locally abundant, R: rare species.

* Mosquitoe species captured under shrubs and undergrowth during the day [34].

Vc: Vector competence (infection rate: IR, transmission rate: TR). CPD50: cytopathic dose 50, SMICLD50: suckling mouse intracerebral 50% lethal doses, PFU: plaque-forming unit.

MV: major WNV vector, CV: candidate vector, PV: potential vector.

For the first criterion, natural infection, 16 mosquito species were found to be WNV-positive under field conditions in Madagascar (Table 1). For the second criterion, no attempt was made to evaluate the vector competence of Malagasy mosquitoes. However, vector competence has been demonstrated in other countries for *Aedes aegypti*, *Ae. albopictus*, *Culex univittatus*, *Cx. quinquefasciatus*, *Cx. pipiens*, *Cx. tritaeniorhynchus*, *Cx. vansomereni*, *Mansonia africana*, and *Ma. uniformis* [25, 38, 48, 70, 115, 134].

The last criterion, field vector-host contact, is well known for these 29 mosquito species. Tantely et al. [127] have listed information on their biology. *Aedes aegypti*, *Ae. albopictus*, *Cx*. *quinquefasciatus*, *Cx. tritaeniorhynchus*, *Cx. univittatus*, and *Ma. uniformis* are abundant in Madagascar but exhibit different host preferences [127]. *Culex quinquefasciatus* and *Ae. albopictus* are highly abundant in urban areas and present across the island [34], while *Cx. univittatus*, *Cx tritaeniorhynchus*, and *Ma. uniformis* occur in rural villages [14, 34, 88, 124]. Only *Ae. aegypti* is currently limited to smaller degraded or more intact forested areas, with the exception of the city of Antsiranana (Diego Suarez), where it and *Ae. albopictus* occur in sympatry [100]. *Culex quinquefasciatus*, *Cx. tritaeniorhynchus*, *Cx. univittatus*, and *Ma. uniformis* have a generalist host-feeding pattern (ornithophily, anthropophily, and mammalophily; Table 1) [127]. *Aedes albopictus* and *Ae. aegypti* are notably anthropophilic and to a lesser extent ornithophilic [34, 123]. These observations highlight the role of *Ae. albopictus*, *Cx*. *quinquefasciatus*, *Cx. tritaeniorhynchus*, *Cx. univittatus*, and *Ma. uniformis* as major vectors of WNV, as well as *Ae*. *aegypti* with local importance.

No information is available on the level of vector competence for eight abundant mosquito species, which include species that are anthropophilic, ornithophilic, and generalist feeders (Table 1), and considered as WNV candidate vectors in Madagascar (*Aedeomyia madagascarica*, *Ae*. *albocephalus*, *Ae*. *madagascarensis*, *Ae*. *circumluteolus*, *Anopheles coustani*, *Cx*. *antennatus*, and *Cx*. *decens*). Wild individuals of four other species, *An*. *brunnipes*, *An*. *maculipalpis*, *An*. *pauliani*, and *An*. *scotti*, tested positive for WNV [34] and should be considered as potential vectors, even though they are rare, but widely distributed in Madagascar. These mosquito vectors are already involved in enzootic circulation of WNV on the island [34, 75].

## Enzootic circulation and WNV maintenance

WNV transovarian transmission or overwintering infected mosquitoes are postulated as the principal means for WNV persistence [3, 11, 39, 86, 135]. Based on the functioning of virus-vector systems and without involving vertebrate hosts, arbovirus maintenance by vertical transmission implicates drought-resistant resting eggs of infected mosquitoes (genus *Aedes*) [108]. During a long inter-epizootic period, this mechanism is improbable in *Culex*, because of egg desiccation, but likely to occur in *Aedes*, as their eggs are resistant [27, 61]. Hence, WNV maintenance by vertical transmission is possible in Madagascar for *Ae*. *aegypti* and *Ae. albocephalus*, taking into account their abundance and field infection with WNV (Table 1) [127]. In the same way, persistence of WNV during the dry season may involve *An. pauliani* and *Ma. uniformis* and at least one *Culex* species that were found WNV-positive in Madagascar (Table 1) [75].

In Madagascar, WNV strains isolated in parts of the island in the late 1980s (1986 and 1988), 2012, and 2013 are genetically closely related [75]. These WNV strains are different from the first isolated strain found in Madagascar in 1978 and those from Africa (western, eastern, central, and southern regions) and the Palearctic (Europe and the Middle East) [41, 75, 90]. These observations suggest a local WNV cycle without new introductions from other countries, as has been reported from South Africa [56].

Some sequence similarities between Russian strains of WNV and those from Madagascar have been proposed [22], though based on rather limited sampling. While this aspect needs further research, it might be associated with Palearctic migratory birds passing through Madagascar (see next section). However, according to Ciccozzi et al. [22] movement might be in the opposite direction and associated with legal and illegal trade of Malagasy birds, amphibians, and reptiles, and possibly the passive transport of infected mosquito vectors through international air flights. Export of *Coracopsis* spp. from Madagascar is known to have occurred between 1991 and 1998 [29].

A hypothesized means of maintenance of WNV in Madagascar is between ornithophilic mosquitoes and local birds, for example in aquatic ecosystems, such as Lac Kinkony and Lac Soamalipo [14, 75, 127], in the western wetland of Madagascar where several species of known mosquito vectors are living in sympatry with a variety of wild non-migratory aquatic and domestic birds. Western wetland bird communities are dominated by herons such as *Egretta* spp. [47]. More specifically, *Ad. madagascarica* is ornithophilic and abundant in these areas, and has frequently been found to be positive for WNV [75, 127] and hence, might maintain WNV in the vector-bird cycle [133]. Clinical examples of WNV infection on the island [78], including a fatal human case [64], may only be the visible “tip of the iceberg” for Malagasy human health and economic threats, given that a fever is too often seen as synonymous with malaria in Madagascar [102]. On the basis of current information, these findings would uphold the hypothesis of WNV enzooticity independent of annual movements of migratory birds passing through Madagascar [56].

Potential involvement of *Egretta* spp. and *Coracopsis vasa* in WNV enzootic circulation might be based on the following criteria: (i) only both wild-bird genera were found to be WNV-positive on the island [34], (ii) they are present throughout Madagascar, and (iii) are often common alongside human activity [47, 143]. In Madagascar, wetland birds breed from October to June, with a peak for many species toward the end of the rainy season in March [47]. In most forest birds, the breeding season coincides with the start of the summer, and many birds have nests and young in December. The biology of *Coracopsis vasa* is well known in the southwest of the island. Ekstrom [29] reported that the genus *Coracopsis* has 15–16 days of egg incubation, with 78% egg hatching success and 35- to 45-day chick-rearing periods. The first egg is laid in October and the last known chick fledges in January.

## Migratory birds in Madagascar

It has been proposed that birds are the primary vertebrate hosts of different arboviruses [1], including WNV [95], at least in part because of their relatively long periods of viremia (see above). Hence, migratory bird species that can cover considerable distances over relatively short periods have been hypothesized to serve as introductory hosts, between their sub-Saharan wintering grounds and Eurasian or Palearctic breeding areas [104]. Further, given that the *Culex* genus is directly implicated as the principal mosquito host responsible for the transmission of WNV and this genus is largely associated with aquatic areas, it is assumed that different forms of waterbirds, comprising a range of different avian orders and families, might be among the most important migrants implicated in the spread of WNV.

Many years ago, Moreau [83] presented details on pathways used by Palearctic migrants returning in the northern spring from sub-Saharan Africa to summer breeding areas. One major flyway funneled into the Upper Nile Valley, leading to the Nile Delta, and northern coast of Egypt, and then directly traversing the Mediterranean Sea or via land across the Middle East. There is no evidence to indicate significant migratory movements from eastern Africa across the Mozambique Channel to Madagascar and then across the southwestern Indian Ocean to Eurasia. Considerable data are available for parts of eastern Africa, the lower Nile Valley, and the Middle East with respect to the diversity and intensity of migratory birds passing through these areas, which in contrast to Madagascar, indicates significant waves and rapid passage of migrants.

The Nile Valley and Delta of Egypt, a zone where epidemic WNV has been documented and where humans show high rates of seropositivity [119], is a large-scale corridor for migratory Palearctic breeding birds that spend the northern winter in different areas of Africa, including a wide assortment of waterbirds belonging to the orders Ciconiiformes, Anseriformes, Gruiformes, and Charadriiformes, as well as a considerable songbird diversity of the order Passeriformes. At least until the late 20th century, the commercial hunting of these birds took place at the level of hundreds of thousands per year, including more than 60 species of waterbirds and a large assortment of songbirds, which were brought alive or dead to town and village markets and sold for human consumption [40]. This commercial trade provides an important interface or amplifying effect between migratory birds with high viremia, mosquitoes, and humans. In contrast, in Madagascar, with the exception of large freshwater lakes such as Lac Alaotra [5], the collection and sale of live waterbirds for bush meat at a commercial scale is largely unknown and mostly concerns non-migratory species.

Another important aspect to consider in these geographical contrasts is bird species diversity. The extant avifauna of Madagascar shows a number of peculiarities, as compared to east Africa, such as relatively low species diversity, with 282 known taxa from the island of which 102 (36%) are endemic [101]. This is low in comparison to 1046 species known from Tanzania and 1008 from Uganda, although the avifauna of these two countries have few endemic species [144].

Another important difference is the magnitude of Palearctic migrants wintering or passing through east Africa as compared to Madagascar. In total, 73 species of breeding Palearctic Passeriformes, many of which have tested positive for WNV during the period of migration [54], are known to winter in sub-Saharan Africa [139]. In contrast, Palearctic migratory passerines are virtually unknown in Madagascar, with less than five species being documented and on few occasions [117], and the often abundant Palearctic migrants known from the African continent of the families Sylviidae, Laniidae, and Emberizidae are completely unknown from the island. Further, only a few Palearctic migratory non-passerines, particularly of the order Charadriiformes, spend the northern winter months in Madagascar [47, 113], and during the migratory season, in comparison to Eastern and North-eastern Africa, both species diversity and absolute numbers are relatively limited [6, 111, 113].

A few other taxa are important to highlight migratory movements between Africa and Madagascar. Two species of falcons, *Falco concolor* and *F. eleonorae*, after exiting their breeding areas pass through eastern Africa [43, 52] and spend the northern winter in Madagascar, the latter species has been shown in another part of its range to have neutralizing antibodies against WNV [36]. Species such as *Ciconia ciconia*, which have been implicated on several occasions in the transmission of WNV between their sub-Saharan and Palearctic breeding grounds [73], are not known to occur in Madagascar. There are four species of non-passerines that nest in Madagascar and spend at least a portion of the non-breeding season in east Africa (*Ardeola idae*, *Glareola ocularis*, *Cuculus rochii*, and *Eurystomus glaucurus*), as well as possible migratory movements of *Phoenicopterus ruber*, *Merops superciliosus*, and different species of ducks between Africa and Madagascar (Steven Goodman, pers. comm.).

The important point is that Madagascar is not within the standard migratory route of breeding Palearctic birds, whether waterbirds or passerines, that winter in sub-Saharan Africa. This greatly reduces the possibility of rapidly passing migratory birds with high viremia reaching the island, which could act as introductory hosts for virulent new strains of WNV. Further, the lack of regular migratory bird flux between Africa and Madagascar would reduce the probability of transmission and the subsequent reintroduction of the virus into locally occurring mosquito species, fitting with the scenario of WNV circulation presented below.

## Scenario of WNV circulation in Madagascar

The main cycle of WNV involves an enzootic cycle (rural cycle) between ornithophilic mosquito vectors and birds (domestic, wild, or both) with continuous transmission, chronic viral infection, and vertical transmission, which act as the maintenance mechanism [10]. This enzootic cycle could lead to an epidemic cycle (urban cycle) when competent vectors (ornithophilic or bridge vectors) and amplifying hosts (humans, horses, and young animals) are contemporaneously present [10, 17]. Possible cycle between birds and mammals could be observed by predation or scavenging [8]. Ticks (Argasidae) could be involved in virus transmission between bird populations when both occur in abundance in the same locality [85].

In Madagascar, a large amount of information is available for the urban cycle [34, 68, 84] and the rural cycle with a sylvatic component being present around or within forested areas [35]. According to the proposed system of mosquito vector categorization cited above, the urban cycle may involve *Cx*. *quinquefasciatus* (Table 1). This species is present in all geographic zones of the island, with a preference for urban environments [34], in tropical areas and Southeast Asia [141], and on the Mascarene Islands [130].

Moreover, the findings of Fontenille et al. [35] in Madagascar highlight two distinct forest cycles involving the dry forest of the Western Domain and the wet forest of the Central Domain. Indeed, among 55 WNV strains isolated from Culicidae between 1982 and 1988, 40 were isolated from mosquitoes, collected within the western dry forests and involving *Ae*. *albocephalus*, *Ae*. *aegypti*, *Ae*. *circumluteolus*, *Ae*. *madagascarensis*, and *Cx*. *tritaeniorhynchus*. Three WNV strains were isolated from *Cx*. *quinquefasciatus* and *Cx*. *univittatus* in a village setting in the Central Highlands and in close proximity to degraded humid forest [35]; *Cx. univittatus* is known to be involved in WNV transmission at this locality [35] and may act as a WNV bridge between forest environments and domestic animal hosts living in nearby villages, or *vice versa*. This species feeds both on the ground and in the upper portions of the forest canopy [55].

Another rural cycle in which WNV is considered endemic, involves aquatic areas, specifically lakes, of the Western Domain [14, 75, 127]. In this cycle, four mosquito species collected in the field, *Ad*. *madagascarica*, *An*. *coustani*, *An*. *pauliani*, and *Ma*. *uniformis*, were frequently found to be WNV-positive [75, 127]; it is assumed that the affinity of *Ad. madagascarica* at Lac Kinkony for shallow areas and lake edge habitats might favor this species as a bridge vector between wild waterbirds and domestic village birds.

Previous work outside of Madagascar has underlined the impact of ecological changes that may enhance local arbovirus outbreaks by disruption of natural enzootic arbovirus cycles [93]. Although currently not documented in Madagascar, virus circulation associated with the domestic bird trade may act as a relay between rural and urban cycles [140]. Dispersal and inter-regional movement of bird pathogens by the commercial bird trade network have already been described in the Lac Alaotra area of eastern Madagascar [19, 105]. In Antananarivo city, *Coracopsis vasa* is seen for sale in Antananarivo near Lac Anosy [29].

Moreover, WNV introduction in areas with infected wild waterbirds, specifically herons and egrets of the order Ciconiiformes, has been suggested in Madagascar [35]. These authors suggested that heronries occurring in populated cites, such as Antananarivo (capital of Madagascar), might be a source of zoonotic reservoirs.

As previously mentioned, Madagascar, as compared to the African and European continents, has notably different dynamics of migratory bird flux between the Palearctic and Afrotropical regions, which might explain the lack of WNV introduction in the island. This aspect combined with current information on genetic variability in the strains of Lineage 2 of WNV [22, 75], where Madagascar forms a separate sub-clade from sequences obtained from African and Eurasian cases, indicates that the island is not in the mainstream of transmission along the African-Palearctic migratory bird routes.

## Risk of WNV transmission in Madagascar

Human exposure via bites by infected mosquitoes is the most important factor in WNV transmission [50, 142]. Mosquitoes that are generalist feeders, rather than with strict host preferences, seem to be more important in WNV circulation [80], probably because they serve as bridge vectors between infected birds and a range of susceptible vertebrate hosts [133]. In Madagascar, the prevalence of WNV infection in humans and animals increases with age, suggesting enzootic circulation and continuous transmission [68, 75, 84]. Moreover, spending more time outdoors and using less personal protection constitute a risk for WNV infection [74], probably due to the longer-term accumulated exposure to bites of diurnal and nocturnal mosquitoes [33, 133].

The increase in local abundance of mosquito vectors coupled with abundance of birds facilitates WNV transmission [73, 103]. However, when the birds are young and susceptible to WNV, there is an upper limit of infection before significant levels of mortality occur, resulting in a reduced possibility of exchange between host and mosquito, and a dramatic decrease in circulating WNV [44]. Entomological results obtained in two longitudinal studies performed in areas of the Central Domain [34, 129] and Western Domain [126] show contradictory results. In the Central Domain, vector populations of mosquitoes are abundant during the rainy season, with *Culex quinquefasciatus* being the most abundant species in urban environments, *Anopheles coustani* and *An. squamosus* in rural and *Cx. pipiens* in forest environments. For the Western Domain, longitudinal data is only available from the western lowland wetlands and the most abundant vector during the dry season is *Aedeomyia madagascarica*. These findings may support the hypothesis that the seasonal patterns of WNV transmission by mosquitoes depend on bioclimatic and environmental factors, which show notable differences in the island’s different bioclimatic domains, as well as with respect to vector diversity.

Bird migration is a major mechanism of WNV dispersion [94, 103] and might be associated with epidemics due to the spread of a virulent lineage with some genetic modification [90] or the passage of migratory birds through infected countries [73, 103]. An important aspect is that infected migratory birds arriving in Madagascar would only be possible reservoirs of WNV for less than seven days, after which decreasing viremia levels would reduce their ability to infect native mosquitoes [59]. The diversity and intensity of migratory birds passing through Madagascar was discussed above and shows some important specificities with respect to east Africa, the zone where WNV was first isolated, and parts of the Nile River Valley and adjacent areas of the Middle East.

The circulation of WNV in different parts of the world is more strongly correlated to temperature than rainfall [112], in contrast to the other arboviruses [125, 129]. In Madagascar, more intense circulation of WNV was observed in western areas, characterized by warmer and drier weather, than in the cooler Central Highlands [68], suggesting that the distribution of WNV could be modulated by varying climatic conditions. Notable fluctuations in annual weather patterns, specifically maximum temperatures, combined with forecasted patterns of long-term climate change constitute important risks in the spread of WNV [31].

The presence of breeding sites favors increased WNV infection [45], principally host-vector contact accentuated by increased populations of mosquito vectors [82, 116]. Furthermore, mosquito species known to be associated with WNV in Madagascar utilize different types of larval breeding sites including terrestrial water accumulation associated with agricultural activity, natural larval habitats associated with terrestrial habitats, and artificial containers [127]. These observations are consistent with the findings of other studies which show augmented populations of WNV mosquito vectors being driven mostly by artificial flooding associated with human activities (cultivation, hunting, and fishing), rather than rainfall, in Camargue, France [9] and flooded basements in Bucharest, Romania [45].

The WNV prevalence and the degree of human-vector contact decrease in areas treated with insecticide [30]. In Madagascar, such vector control programs intend to target mosquitoes transmitting malaria with indoor residual spraying (IRS) and the use of bed nets (insecticide-treated nets (ITNs) and long-lasting insecticide-treated net (LLIN)) [7]. However, as described by Geissbühler et al. [37], ITNs might offer higher protection against exposure to endophagic rather than exophagic mosquito species (Table 1). Indeed, these IRS and/or LLIN interventions may result in a reproductive advantage for those mosquitoes that opportunistically feed outdoors as observed in Equatorial Guinea [107]. To date, no action has been undertaken in Madagascar to reduce outdoor mosquito bite protection among humans.

## Perspectives

Feeding behavior, specifically the host choice of mosquito vectors of WNV, is influenced by environmental and spatial factors, habitat, and differences in the biology of various bird groups at different taxonomic levels [58, 81, 121, 132]. Despite some existing studies on the feeding behavior of different vector species [14, 34, 127], no information on WNV circulation in ornithophilic mosquito species associated with native forest-dwelling birds is available for Madagascar and few details are known in general for the Old World tropics. Further studies on the vertical distribution of mosquito vectors and their feeding behavior are needed in forested environments of Madagascar.

As found in areas of the New World, periods of mosquito and bird abundance are related to high infection rates in mosquitoes [89]. In general, little information on monthly densities of mosquitoes is available for Madagascar, with the exception of studies in the Central Highlands. In the western wetland of Lac Kinkony [34, 126, 128, 129], combined studies of vector populations overlaid with bird population dynamics should be undertaken, specifically focusing on lowland aquatic environments.

As the main affected ruminants in several countries are horses [67], research needs to be conducted on mosquito WNV transmission to the island’s equine population, which should also include serological studies.

To date, no attempts have been made to evaluate insecticide resistance of arbovirus vectors in Madagascar. However, a control program cannot succeed without adequate information on insecticide resistance in the vector, given that some WNV vectors (*Aedes albopictus* and *Cx. quinquefasciatus*) are able to develop resistance to several compounds by expressing multiple resistance mechanisms [130]. Finally, given the pathogenicity of the local WNV and high incidences of human infection [64, 68, 78], regular surveys of WNF should be conducted.

## Conclusion

In Madagascar, natural populations of 16 species of mosquitoes were found to be WNV-positive. *Aedes albopictus*, *Culex quinquefasciatus*, *Cx. tritaeniorhynchus*, *Cx. univittatus*, and *Mansonia uniformis* can be considered as major WNV vectors. Nine mosquito species belonging to the *Aedeomyia*, *Aedes*, *Anopheles*, *Culex*, and *Mansonia* genera should be considered candidate vectors, while four species are designated as potential WNV vectors. WNV circulation seems to occur in three types of epidemiological cycles: urban, forest, and wetland. *Aedeomyia madagascarica* could maintain WNV through a vector-bird cycle around lakes where WNV is endemic. However, in Madagascar conclusive data on bird-vector contact, including domestic fowl, resident and migratory birds, particularly waterbirds, are lacking and it is not possible to characterize how WNV is maintained and circulated within a vector-bird cycle. The flux of Eurasian migrant birds in the northern spring passing through Madagascar en route from east Africa to the Palearctic is very limited. This situation greatly reduces the possibility of these animals carrying and transmitting WNV to the island. The vector capacity of mosquitoes, related to the biology and dynamics of mosquitoes and their interactions with local birds, is a promising direction for future research to understand the local maintenance of a WNV cycle in Madagascar.
